# An investigation of factors identified at birth in relation to anxiety and depression in old age: the Hordaland Health Study (HUSK)

**DOI:** 10.1186/1471-244X-13-136

**Published:** 2013-05-10

**Authors:** Jens Christoffer Skogen, Robert Stewart, Arnstein Mykletun, Marit Knapstad, Simon Øverland

**Affiliations:** 1 Department of Health Promotion and Development, Faculty of Psychology, University of Bergen, Bergen, Norway; 2 Division of Mental Health, Department of Public Mental Health, Norwegian Institute of Public Health, Bergen, Norway; 3 King’s College London (Institute of Psychiatry), London, UK

**Keywords:** Foetal origins of disease, Anxiety, Depression, Old age, Early life factors, Birth weight

## Abstract

**Background:**

Although life course influences have long been recognised in affective disorder, little is known about the influence of early life factors on late life anxiety and depression. The aim was to investigate the extent to which birth measures, maternal health and family circumstances were associated with symptoms of anxiety and depression in late life.

**Methods:**

A retrospective cohort study was constructed from a cross-sectional survey sample of community residents aged 72–74 years, 406 of whom had traceable birth records. Cases and controls for late life anxiety and depression were defined applying standard cut-offs to the Hospital Anxiety and Depression Scale. A range of measures and circumstances were extracted from birth records blind to survey data and compared in age- and gender-adjusted models.

**Results:**

There were no differences in any anthropometric measure in either case control comparison. Case-level anxiety and depression were both associated with significantly lower maternal age. Late-life anxiety was additionally associated with smaller maternal pelvic size and the mother’s condition being rated as poor at birth/discharge. Late-life depression was associated with a lower status paternal occupation.

**Conclusions:**

There was no evidence for a substantial influence of early life size on late life affective disorder. However, there was some evidence in secondary analyses for an enduring influence of the family’s socioeconomic environment and maternal health.

## Background

Anxiety and depression are highly prevalent disorders among older adults [1,2] and, with increasing life expectancy, an area of growing clinical and public health significance [3]. A recent review of risk factors for anxiety and depression in old age highlighted personality traits, inadequate coping strategies, previous psychopathology, qualitative aspects of social networks, stressful life events and female gender as important risk factors [2]. Despite considerable overlap in risk profiles for anxiety and depression, some risk factors appear more relevant for depression specifically, including chronic diseases, poor self-perceived health, functional disability, shrinking social networks and being unmarried [2]. Facing this combination of proximal and distal risk factors, a life-course perspective including risk factors originating from the early-life environment [4], is potentially indicated.

The developmental origins of adult health and disease (DOHAD), originally known as “the Barker hypothesis”, is commonly applied as the framework for understanding empirically observed links between processes at prenatal stages and adult chronic disease [5,6]. The DOHAD-hypothesis suggests that prenatal risk factors, from intrauterine environmental exposures, affect the foetus irreversibly during specific developmental periods. Thus, according to the hypothesis, the risk of specific diseases such as coronary heart disease and non-insulin dependent diabetes in adult life, will be elevated across the life-span for those who are exposed to the relevant risks in foetal life [7]. A key practical challenge in research on the hypothesis is to attain information about the offspring’s development during pregnancy. Anthropometric measures such as birth weight, birth length and head circumference are routinely, and often reliably recorded, and therefore often used as proxy measures [8].

The DOHAD-hypothesis has also been applied to investigate the aetiology of mental disorders, proposing that prenatal factors can modify risk [9]. Low birth weight has been linked to increased risk of suicide [10,11], schizophrenia [12], and autism [13], as well as with anxiety and depression [4,9,14-27]. In 2007, Alati and colleagues [9] reported an inverse linear association between birth weight and depressive symptoms at age 21 in females, but not in males. Wiles and colleagues found the same pattern in relation to risk for psychological distress among middle-aged individuals of both genders [20].

To our knowledge, only three studies have investigated the impact of early life factors and late-life mental health. Thompson and colleagues found that foetal undernutrition predisposed men, but not women, to depression at age 68 [14]. A recent Swedish study found that women born at a lower gestational age and those born with a birth weight less than the median, were more likely to have reported lifetime depression with a follow-up of 92 years of age [25]. As the latter study investigated the risk of life-time depression, however, the finding may not reflect an enduring risk of gestational age and birth weight for depression into old age. On the other hand, Gale and colleagues found no association between birth weight and anxiety and depression at age 64-79 as reported on the Hospital Anxiety and Depression Scale [28] in five different cohorts [4]. Other studies in younger populations have also failed to identify associations between foetal or very early life factors and mental disorders in adolescence, early adulthood and mid-life [29-34], leaving the question open for debate [27]. A recent systematic review investigating the foetal origins of depression included 18 studies and compared those of low birth weight (<2500g) to those of normal birth weight (>2500g) [35]. The authors found evidence for a weak association (random effects estimate of OR 1.15 (CI95% 1.00-1.32)) between low birth weight and later depression or psychological distress, but also indications of publication bias. Furthermore, studies of associations between perinatal status and later mental disorders have been criticized for relying too much on birth weight as the indicator of the foetal environment [4] and this may contribute to the heterogeneous results, in addition to different lengths of follow-up. It has also been argued that associations may be more prominent in females and less identifiable in male-only cohorts [36].

In summary, there is some evidence suggesting an association between foetal and early origins of anxiety and depression in adulthood. This is however not consistently supported in the literature, with study design differences and elements of publication bias suggested as possible reasons for heterogeneity. Compared to research on depression in old age, anxiety disorders have received very little attention [37], despite similar prevalences to depression in old age [38] and high levels of co-morbidity with depression [2,38].

Taking advantage of a birth record archive in an area covered by a cross-sectional survey of older people’s mental health in Bergen (Norway), we constructed retrospective cohort study to investigate the relationship between the early life environment and late life anxiety and depression. This allowed for a follow-up from the 1920s to the 1990s. Primary hypotheses were that smaller anthropometric measures would be associated with case status for both syndromes. Secondary analyses investigated other information reflecting parental health and early life socioeconomic status.

### Cohort context – characteristics of Bergen in the early 20th century and life expectancy

During the late 19th century and early 20th century, Bergen city expanded geographically and qualitatively from a semi-rural city to one with more modern characteristics. Primary industry which had dominated, gave ground to expanding secondary and tertiary industry [39]. This change in industry was mostly due to growing production and manufacturing, but also due to an increase in commerce, shipping, transport and service sectors [39]. As a consequence of this, three social classes began to dominate in Bergen during the same period, upper (bourgeoisie), middle and lower, with large differences in income, housing standard and diet. The upper class was characterised by industry proprietors, importers, wholesale dealers and financers. The middle class consisted primarily of merchants, craftsmen and officials, while the lower class comprised regular workers or artisans [39]. During 1925 and 1927 the life expectancy at birth in Norway was approximately 67 years for males, and 74 years for females [40].

## Methods

### Study population

The sampling frame for this study comprised all 3,341 (77% of the invited) participants of the old age cohort of the population-based Hordaland Health Study (HUSK) which has been previously described [41]. In summary, all residents of Bergen city or neighbouring areas born between 1925 and 1927 of a previously established study population were invited to participate in a general physical examination and to complete questionnaires on socio-demographic status, general health and health-related behaviour. HUSK was conducted from 1997 to 1999 as a collaboration between the National Health Screening Service, the University of Bergen and local health services.

In this study, we sought to link as many members as possible of the HUSK sample to their birth records in order to construct a nested retrospective cohort study within the linked subgroup, comparing historically recorded information at birth between participants with/without a mental disorder in late life. In the Norwegian Population Registry, all inhabitants of Norway are registered with a personal identification number. Using this individual identifier, the names (and maiden name for females), date of birth, place of birth and parents’ names (if available) of HUSK participants were retrieved. This information was used to trace the participants born in Bergen to the public maternity ward (“Fødestiftelsen i Bergen”) birth records located at the Regional State Archives of Bergen. In the second decade of the 20th century, about one quarter of all births in the Bergen area took place in the official maternity ward (personal communication, State archivist). The portion of deliveries taking place at hospitals increased steeply when the new Women’s Clinic (“Kvinneklinikken”) was inaugurated in 1926, replacing the old maternity ward. The relevant birth records for the present study were those detailing births between 1st of January 1925 and 31st of December 1927. These records contain detailed information about the pregnancy, the birth and the mother’s health recorded by midwives and obstetricians during the hospital stay. The Women’s Clinic in question was the main teaching facility for midwifes at the time, and the records were integral to the training, and are therefore considered to be of high quality [42].

### Early life factors – information obtained at birth, 1925–27

The available birth records in the Regional State Archives of Bergen were viewed and coded by researchers blind to all measurements made in HUSK. The following information was abstracted from each record (directly copying original information unless stated otherwise): birth weight (kg), birth length (cm), head circumference (cm) at birth, ponderal index (PI; calculated from weight divided by the third power of length), mother’s pelvic size (cm), mother’s age (years), and gestation (weeks). The following binary variables were derived from individual free text fields: any recorded disease in the mother, family history of coronary heart disease (yes/no) and tuberculosis (yes/no), the state of mother’s teeth (poor/good), complications during birth (yes/no), mother’s condition after birth (poor/good; based on the midwives description of the mother (e.g. fatigued, pale, swollen and feverish)), mother’s condition at discharge (poor/good; based on the midwives description of the mother (e.g. pale, swollen and feverish), and indicators of socioeconomic status included marital status (married/unmarried), father’s occupation (unskilled/manual vs skilled manual/non-manual), and type of payment for the hospital stay (health insurance/not insurance).

### Anxiety and depression – follow-up in HUSK at age 72–74

Cases and controls were defined according to the presence of anxiety and depressive symptoms in late life, ascertained in HUSK participants using the Hospital Anxiety and Depression Scale (HADS) [28]. HADS assesses anxiety (HADS-A) and depression (HADS-D) on two separate subscales, each consisting of seven questions. The HADS has been used extensively in previous community-based research and is considered a reliable and valid screening tool, with little measurement variance as a function of age in the general population [43,44]. As HADS was developed for a hospital setting, no somatic symptoms of anxiety or depression are included, which renders it especially suitable for assessment in an old age population where significant comorbidity with physical health conditions is anticipated [45]. In accordance with previous studies, the mean score for all individuals with five or more valid responses on each subscale was computed [44]. We employed the recommended cut-offs to define caseness (both ≥8), which have been found to yield an optimal balance between sensitivity and specificity (both 0.8) [43]. Applying these cut-offs, two dichotomous variables for case-level anxiety and depression were created, where the controls for anxiety could include case-level depression and vice versa. Our findings are equivalent when employing a control group restricted to only non-cases or HADS as a continuous measure (data not shown).

### Additional demographic information – follow-up from HUSK at age 72–74

To assess potential demographic differences between the participants we were able to trace and the remaining HUSK participants, we obtained gender, self-reported level of educational attainment and general health from HUSK. Level of educational attainment was divided into “compulsory only” and “post-compulsory”, while general health was divided into “poor” and “good”.

### Statistical analyses

Of the 3,341 HUSK-participants [41], we were able to trace 480 to their birth records. Out of these, 406 (84.6%) had five or more valid responses on each HADS subscale (comparable to the rest of the HUSK-participants) allowing late life case/control status to be ascertained and constituted the total study population. The sample was described, HUSK participants with traceable birth records were compard to the remainder of the sample, and the traced with and without valid HADS-subscales were compared in relation to the exposures. For primary analyses, previous exposure status was compared between cases and controls using age- and gender-adjusted linear regression analyses for continuous exposures and similarly adjusted logistic regression models for categorical exposures. In order to assess adverse conditions in the womb, the association between birth weight and case-level anxiety and depression was adjusted for gestational age in addition to age and gender. In total, 18 potential exposures were investigated in relation to each HADS subscale. Although not entirely uncontroversial, considering the explorative nature of the study and the limited sample size, we chose not to adjust for multiple comparisons. For the significant associations, we also performed a secondary analyses adjusting for educational attainment and self-reported general health at participation in HUSK. Stata version 11.0 [46] was employed for all analyses presented in this paper.

Using the software G*Power version 3.1.3 [47], a *post hoc* power analysis indicated that we would be able to detect a small to medium effect size for mean group differences (Cohen’s *d* of 0.39) and binary associations (Cohen’s *w* of 0.18), given a power of 80% for the main HADS-A and HADS-D analyses [48-51].

### Ethics

The data in HUSK was collected in accordance with ethical standards required by the regional ethical board of Committees for Medical and Health Research Ethics in Norway (REC). The permission to collect and store the data from HUSK was given by the Norwegian Data Inspectorate. All participation in HUSK was voluntary, and all potential participants received written information about the project before they met for examination. The participants gave their written statements of informed consent, including the specific consents to use information from HUSK in health research and to link this information with other relevant data sources. The participants also gave their written statement that they were informed that no specific time-limit was set for the storage of data. This specific study was reviewed and approved by REC. The current study adheres to the standards of the declaration of Helsinki.

## Results

No differences between the HUSK-participants with birth journal information and participants without were found regarding gender (p=0.814), self-reported health (p=0.706), case-level anxiety (p=0.531) or case-level depression (p=0.621). However, participants we were able to trace had a significantly higher educational attainment (p=0.025) than the untraced. The proportion of participants with case-level anxiety and case-level depression in the traced sample was 14.3% and 9.4%, respectively. No differences were found on exposures obtained at birth between those with valid HADS subscales (n=406) and those without (n=74), all p-values ranging between 0.087-0.990. Out of the 406 HUSK-participants included in the present study, 54.2% (CI95% 49.3-59.0) were female, and the mean age was 72.3 (SD: 0.9) years. The sample-characteristics obtained at birth are summarized in Table 1.

**Table 1:** Sample characteristics at birth obtained from medical records

	**N=**	**Mean**	**Proportion (%)**	**Standard deviation**
Birth weight (kg)	406	3.47	-	0.51
Birth length (cm)	406	50.30	-	2.04
Head circumference (cm)	400	34.47	-	1.66
Ponderal Index	406	2.65	-	0.22
Mother’s pelvic size (cm)	382	26.05	-	1.30
Mother’s age (years)	406	29.24	-	5.73
Parity	406	2.57	-	1.94
Gestational time (weeks)	406	39.67	-	1.18
Gender (% female)	406	-	54.2%	-
Mother’s condition after birth (% poor)	406	-	9.9%	-
Tuberculosis in family (% yes)	406	-	10.8%	-
CVD in family (% yes)	406	-	11.6%	-
Mother’s condition at discharge (% poor)	406	-	23.2%	-
Complications birth (% yes)	406	-	9.6%	-
Father’s occupation (% manual/unskilled)	406	-	59.4%	-
Unmarried (% yes)	406	-	3.9%	-
Teeth lower jaw (% poor)	394	-	57.9%	-
Payment (% not insurance)	289	-	57.1%	-
Number of diseases (%>1)	406	-	30.0%	-

In the main analyses, none of the anthropometric measures at birth, such as weight and length, differed significantly between cases and controls in either comparison (Table 2), and out of the 36 bivariate associations investigated, only 6 (16.7% CI95% 3.9%-29.5%) were statistically significant (Tables 2 and 3). For continuous and ordinal exposures, both case-level anxiety and depression were associated with lower mother’s age (p=0.027 and p=0.036, respectively), and case-level anxiety was associated with a smaller maternal pelvic size (p=0.017). For binary exposures, case-level anxiety was associated with an increased odds of the mother’s condition after birth or at discharge being rated as poor (p-values 0.001 and 0.012 respectively). Case-level depression was associated with an increased odds of a lower occupational status for the father (p=0.029). There were no substantial differences between the crude model and age- and gender-adjusted model for any of the associations investigated, and modelling the HADS-subscales as continuous outcomes also yielded similar results as did restriction of controls to those with neither anxiety nor depression (data not shown). For the significant results, adjusting for educational attainment and self-reported general health in HUSK only minimally changed the estimates (data not shown).

**Table 2:** Associations between late life anxiety and depression case status and continuous or ordinal exposures recorded at birth

	**Mean (SD)**		**Age- and gender-adjusted mean difference (95% CI)**	**Mean (SD)**		**Age- and gender-adjusted mean difference (95% CI)**
	**Case-level anxiety**	**Controls**		**Case-level depression**	**Controls**	
Birth weight (kg)^a^	3.38	3.48	−0.06	3.42	3.48	−0.05
	(0.48)	(0.51)	(−0.18,0.06)	(0.62)	(0.50)	(−0.20,0.09)
Birth length (cm)	49.92	50.36	−0.21	50.00	50.33	−0.41
	(2.15)	(2.02)	(−0.78,0.35)	(2.49)	(1.99)	(−1.08,0.25)
Head circumference^b^ (cm)	34.28	34.50	−0.07	34.34	34.48	−0.23
	(1.52)	(1.68)	(−0.54,0.39)	(1.93)	(1.63)	(−0.78,0.32)
Ponderal Index	2.64	2.65	−0.01	2.64	2.65	−0.00
	(0.23)	(0.22)	(−0.07,0.05)	(0.27)	(0.22)	(−0.08,0.07)
Mother’s pelvic size^c^ (cm)	25.65	26.11	−0.44^*^	25.89	26.06	−0.22
	(1.28)	(1.29)	**(−0.82,-0.06)**	(1.05)	(1.32)	(−0.67,0.23)
Mother’s age (years)	27.69	29.50	−1.97^*^	27.37	29.43	−2.14^*^
	(5.85)	(5.67)	**(−3.59,-0.35)**	(5.77)	(5.70)	**(−4.07,-0.22)**
Parity	2.26	2.63	−0.40	2.24	2.61	−0.39
	(1.38)	(2.02)	(−0.95,0.15)	(1.72)	(1.96)	(−1.05,0.26)
Gestation (weeks)	39.63	39.67	0.00	39.62	39.67	−0.05
	(1.14)	(1.18)	(−0.33,0.34)	(1.14)	(1.18)	(−0.45,0.34)
*N*	58	348	406	38	368	406

^*^p < 0.05, ^**^p < 0.01, ^***^p < 0.001.

^a^Birth weight was also adjusted for gestational age.

^b^N=400 (Males N=183 and females N=217).

^c^N=382 (Males N=174 and females N=208).

**Table 3:** Associations between case-level anxiety and depression, and dichotomous exposures in the birth journals

	**Case-level anxiety (%)**	**Controls (%)**	**Age- and gender-adjusted OR (95% CI)**	**Case-level depression (%)**	**Controls (%)**	**Age- and gender-adjusted OR (95% CI)**
Mother’s condition after birth (% poor)	22.4	7.8	5.29^***^	7.9	10.1	1.00
			**(2.30-12.19)**			(0.28-3.54)
Tuberculosis in family (% yes)	8.6	11.2	0.69	5.3	11.4	0.42
			(0.25-1.85)			(0.10-1.81)
CVD in family (% yes)	10.3	11.8	0.93	2.6	12.5	0.18
			(0.37-2.34)			(0.02-1.34)
Mother’s condition at discharge (% poor)	36.2	21.0%	2.05^*^	34.2	22.0	1.75
			**(1.11-3.77)**			(0.85-3.60)
Complications birth (% yes)	10.3	9.5	1.04	18.4	8.7	2.35
			(0.41-2.64)			(0.95-5.82)
Father’s occupation (% unskilled/manual)	55.2	60.1	0.75	76.3	57.6	2.36^*^
			(0.42-1.32)			**(1.08-5.15)**
Unmarried (% yes)	8.6	3.2	2.50	7.9	3.5	2.23
			(0.81-7.75)			(0.59-8.42)
Teeth lower jaw ^a^ (% poor)	57.1	58.0	0.89	52.6	58.4	0.83
			(0.49-1.60)			(0.42-1.64)
Payment ^b^ (% not insurance)	59.1	56.7	1.08	62.5	56.4	1.21
			(0.54-2.16)			(0.55-2.66)
Number of diseases (>1)	25.9	30.8	0.76	31.6	29.9	1.11
			(0.40-1.47)			(0.53-2.31)
*N*	58	348	406	38	368	406

^*^p < 0.05, ^**^p < 0.01, ^***^p < 0.001.

^a^N=394 (Males N=181 and females N=213).

^b^N=289 (Males N=135 and females N=154).

## Discussion

### Main findings

Many previous studies have found an association between early life factors and later mental health problems, but most of these have been limited to follow-up into childhood, early adulthood or middle-age. In this study investigating risk factors present at birth in relation to anxiety and depression in old age, no associations were found for the anthropometric measures of principal interest. However, there was evidence in secondary analyses for associations with factors that might indicate worse maternal health and/or socioeconomic status.

### Strengths and limitations

This study had several strengths. Having access to birth records from the 1920s and the possibility to link this information to a population-based health survey in the late 1990s enabled examination of an interval between exposure/outcome status in the range of 72–74 years. The exposure source contained detailed, accurate and rich anthropometric measurements (such as birth weight in kilograms with two decimal places), as well as information about maternal health and circumstances, the birth process and the early post-natal period. This information is unlikely to be biased in any particular direction. The birth records were carried out as a key element in the education of midwives under the supervision of the head physician. The HUSK study included a validated and often used screening inventory for symptoms of anxiety and depression. Furthermore, both men and women were represented in our sample, enabling identification of any confounding by gender.

Limitations include the relatively small sample size and proportion of the HUSK-participants that could be traced back to their birth records. There are several reasons for this: the birth records were only available for a subgroup as not everyone who participated in HUSK was born in the Bergen area, and some born at home or at other hospitals. Based on a conservative estimate, at least one-third of the HUSK sample would not be within the catchment area of the regional hospital at the time of birth. The small sample size reduces our ability to detect potential small but meaningful effects. A recent meta-analysis investigating the association between low birth weight and depression later on found only small effects [35] – and given the lack of precision in our estimates such small effects may not be detectable in our study. In previous DOHAD-studies which have used birth cohorts to trace individuals in their adulthood (i.e. forward tracing), the identified proportions of the original sample are sometimes as low as 2-5% [52]. The low traceability and small sample size constitute central limitations to our study, and warrants caution with regards to the interpretation and generalisability of the present study. For birtweight in particular, the small sample size precluded any investigation of low birth weight (<2.5 kg) or high birthweight (>4.5 kg) per se, which is associated with later psychopathology in some studies [53,54]. Due to long follow-up between the exposures and the outcomes, survival and selection effects are likely to influence the nature of the sample overall, including a healthy survivor effect [55], and non-participation bias [56] rendering the participants in our sample healthier than those from other studies in younger age groups. It is, however, unlikely that such effects would substantially influence the exposure-outcome associations of interest since both cases and controls were survivors and drawn from the same source population. Also we did not find any systematic differences in gender, self-reported health or HADS-scores between the participants we were able to trace, and the remainder of the HUSK-participants, suggesting reasonable generalisability beyond the analysed sample.

The outcome measure, HADS, is a screening instrument for anxiety and depression, primarily designed to measure these syndromes in a general hospital population. In the age group in this study, a measurement which specifically focuses on cognitive rather than somatic symptoms of anxiety and depression may have benefits in avoiding over-ascertainment due to physical health problems, which are relatively common in this age group. The HADS has been found to have good case-finding properties in a GP setting [57] although remains a screening tool rather than a diagnostic instrument. Furthermore, the cutoff ≥8 have been shown to provide the optimal balance between sensitivity and specificity in previous studies, but it is not clear whether this cutoff is optimal in older age groups. Some studies have suggested a cutoff ≥11 in older populations [45], but we chose to use 8 due to limitations in sample size. In relation to the number of associations investigated (36 in total), we decided not to adjust for multiple comparison, which is in line with some recommendations [58]. Applying Bonferroni adjustments would reduce the number of significant associations to one (case-level anxiety associated with mother’s condition after birth) but not change our conclusions dramatically. Other limitations were that information on maternal health and family circumstances as from the birth records was relatively crude, and gestational time had relatively little variance (with only 8.9% delivered before 40 weeks). These may have obscured some associations, although systematic bias is unlikely since the information gathering in HUSK was unlikely to have been influenced by birth circumstances and the information from the birth records was historic and transcribed blind to case status.

### Interpretation of our findings

Out of the three previously published papers on the relationship between factors present at birth and anxiety and depression in old age [4,14,25], only one has included both genders [4]. The authors of the latter also employed HADS as the outcome measure, and found no association between birth weight and anxiety or depression in old age across five different cohorts. They did not investigate other factors present at birth, and speculated that birth weight might be a too crude marker for fetal neurodevelopment [4]. We expanded on this potential limitation, and included several anthropometric measures, as well as factors like mother’s condition, maternal familial history of disease and socioeconomic status. Interpretation of our secondary analysis should be done with caution, but the present results are indicative of possible continuation of socioeconomic disadvantage and an adverse impact for the offspring if the health in the mother was judged as poor during the hospital stay. Younger maternal age might lead to less experienced upbringing, or might reflect differences in socioeconomic position. The lack of association with parity at birth, however, does not suggest that the inverse association between case-level anxiety and depression with maternal age is because of participants’ positions in their sibships. Associations with smaller pelvic size might be explained by poor nutrition during the mother’s childhood, which in turn could negatively affect the development of the placenta and fetus in late pregnancy [59], although it might also increase the risk of a difficult labour. Worse condition of the mother is of interest as this could reflect a variety of issues, including post-natal disorder, somatic illness or lower socioeconomic position. There was also some suggestion in our findings that socioeconomic markers were more associated with depression, and physical health markers more with anxiety.

Our results are, however, not in line with the finding from the Swedish study where both low birth weight and shorter gestational age at birth were independent risk factors for life-time depression among women [25], although this may be related to the previously mentioned low variance in gestational age in our sample. There are other reasons as to why few of the indicators were associated with anxiety and depression in old age. First, it is possible that factors identified at birth are more important in early or mid-life, but become less relevant as the time between exposure and outcome increases. Instead, other more proximal factors become stronger predictors of anxiety and depression in old age, such as social environment [60] stressful life-events and loss of function [2,4]. Second, is possible that other measures of the foetal environment than those used here would be better indicators of any true link to anxiety and depression in old age, such as the size and shape of the placental surface [61].

## Conclusions

As the life expectancy in many societies has increased, many individuals will live longer, and the pool of older adults with mental disorders will increase. Despite this development, less attention has been directed towards identifying risk factors for late life mental disorders, especially anxiety, than in younger age groups [38,62]. A better understanding of risk factors across the life-span, also during early life and foetal development, is needed, as this might help to increase identification and treatment of mental disorders in old age [2]. Our findings indicate that there is only a weak association between status at birth and anxiety and depression in old age, and it might be that the effects of these risk factors are restricted to early or mid-life mental disorders. Although there was no clear evidence of any impact from educational attainment and physical health later in life for the investigated associations, a better perspective might be to adopt a life-course perspective or developmental origins of health and disease perspective, where foetal and early life-factors are considered in conjunction with physical and social exposures across the whole life-span [63-65]. This will allow for the exploration of early life factors’ potential impact on and ability to modify later exposures related to the development of symptoms of anxiety and depression.

## Competing interests

The authors declare that they have no competing interests.

## Authors’ contributions

JCS, AM and RS were responsible for the conception this study, and the study design was developed by JCS, AM and RS. Analyses were carried out by JCS under supervision of RS and SØ, and manuscript preparation was led by JCS in cooperation with RS, SØ and MK. JCS, SØ, MK, AM and RS were all involved in the interpretation of data, drafting the article and approval of the final manuscript. JCS is the guarantor for the study.

## Pre-publication history

The pre-publication history for this paper can be accessed here:

http://www.biomedcentral.com/1471-244X/13/136/prepub
